# Quantitative measurements of HER2 and phospho-HER2 expression: correlation with pathologic response to neoadjuvant chemotherapy and trastuzumab

**DOI:** 10.1186/1471-2407-14-326

**Published:** 2014-05-08

**Authors:** Huan Cheng, Yalai Bai, William Sikov, Natalie Sinclair, Veerle Bossuyt, Maysa M Abu-Khalaf, Lyndsay N Harris, David L Rimm

**Affiliations:** 1 Department of Pathology, Yale University School of Medicine, 310 Cedar Street, PO Box 208023, New Haven, CT 06520-8023, USA; 2 Department of Internal Medicine, Warren Alpert Medical School of Brown University, Providence, RI 02903, USA; 3 Department of Internal Medicine, Yale University School of Medicine, New Haven, CT 06510, USA; 4 Division of Hematology and Oncology, Case Western Reserve School of Medicine, Cleveland, OH 44106, USA

**Keywords:** Immunohistochemistry, Immunofluorescence

## Abstract

**Background:**

Preoperative therapy with chemotherapy and the HER2-targeted monoclonal antibody trastuzumab is valuable for patients with large or locally advanced HER2-positive (HER2+) breast cancers but traditional methods of measuring HER2 expression do not accurately stratify patients for likelihood of response. Quantitative immunofluorescent approaches have the potential to provide a mathematically continuous measure of HER2. Here we seek to determine whether quantitative measurement of HER2 or phospho-HER2 correlates with likelihood of response to trastuzumab- containing neoadjuvant therapy.

**Methods:**

We evaluated core biopsy samples from 27 HER2+ breast cancer patients enrolled in a preoperative clinical trial using trastuzumab, nab-paclitaxel and carboplatin combination therapy (BrUOG BR-211B (NCT00617942)). Tumor core biopsies were taken before initiation of treatment and 9–13 days after patients received "run-in" doses of either single agent trastuzumab or nab-paclitaxel. The AQUA method of quantitative immunofluorescence was used for analysis of *in situ* protein expression. Patients then received 18 weeks of treatment, followed by surgery to assess pathologic response to the neoadjuvant regimen.

**Results:**

A HER2 score of 2111 by AQUA analysis has been shown to be equivalent to HER2 3+ by immunohistochemical staining in previous studies. Of 20 evaluable patients, 10 cases who achieved a pathologic complete response (pathCR) with neoadjuvant treatment had a mean HER2 level of 10251 compared with 4766 in the patients without pathCR (p = 0.0021). Measurement of phospho-HER2 showed no difference in pathCR vs non-pathCR groups. In 9 patients who had HER2 levels repeated after a single treatment with trastuzumab there was no evidence of a reduction in the HER2 or phospho-HER2 levels following that exposure.

**Conclusions:**

High levels of HER2 are associated with achievement of a pathCR in the preoperative setting, while levels of Phospho-HER2 were not predictive of response. This data suggests that accurate measurement of HER2 may help determine the likelihood of response in the pre-surgical setting. Further validation in larger cohorts is required, but this pilot data shows the feasibility of this approach.

## Background

Human epidermal growth factor receptor 2 (HER2) is amplified or over-expressed in around 20% of breast cancer cases, and the amplification of HER2 is usually associated with worse prognosis [1-3]. Trastuzumab, a humanized monoclonal antibody, was the first drug developed to target HER2 amplified breast cancer. The addition of trastuzumab to cytotoxic chemotherapy showed significant improved time to progression, overall response rate, response duration and overall survival (OS) in advanced HER2-positive breast cancer (HER2+), resulting in FDA approval of the drug in 1998 [4]. In 2006, FDA approval was extended to use of the drug in combination with chemotherapy in the adjuvant setting in early stage HER2 positive breast cancer [5,6]. There are several proposed mechanisms of action. Some studies suggest that the drug disrupts ligand-independent transmembrane signaling triggered by the formation of HER2:HER2 homodimers or HER2:HER3 heterodimers, thereby diminishing Akt pathway activation, which ultimately leads to cell apoptosis [7]. Other mechanisms of cytotoxicity, including activation of antibody-dependent cell-mediated cytotoxicity (ADCC) [8] or blocking the cleavage of HER2 extracellular domain [9] have also been described.

Pre-surgical or neoadjuvant chemotherapy is standard therapy for inflammatory and locally advanced breast cancer. The addition of trastuzumab to chemotherapy in the pre-surgical setting in HER2+ patients has been tested in several phase II studies [10-14], with pathological complete response (pathCR) rates ranging from 18% to 47%. A phase II-III randomized pre-surgical trial conducted by the M.D. Anderson Cancer Center shows significant improvement in the pathCR rate with the addition of trastuzumab to chemotherapy [15]. The NOAH (NeO-Adjuvant Herceptin) trial is a phase III trial that evaluated the addition of trastuzumab to anthracycline- and taxane -based chemotherapy for HER2-positive patients with locally advanced or inflammatory breast cancer. Again, the addition of trastuzumab resulted in an increased pathCR rate, which translated into improved event free survival and OS [16]. In the phase III GeparQuattro trial, evaluating the effect of the addition of capecitabine to epribubin/cyclophosphamide/docetaxel regime, 445 HER2+ patients among 1509 patients with operable or locally advanced tumors were also given trastuzumab. The pathCR rate in the HER2+ subset was 31.7% [17]. Taken together, these trials suggest that the addition of trastuzumab to pre-surgical chemotherapy significantly improves outcomes in HER2+ breast cancer patients.

Although the use of trastuzumab as part of the pre-surgical regimen for breast cancer has increased, a uniform clinical benefit of trastuzumab in combination with chemotherapy is not observed. The likelihood of achieving a pathCR in this cohort is higher in trastuzumab treated patients with hormone receptor- negative HER2+ cancers compared to hormone receptor-positive HER2+ cancers [18]. Other groups have used mRNA measurements to predict pathCR. For example Denkert and colleagues found that quantitative assessment of mRNA for ESR1 and HER2 can predict pathCR [19]. Still, there remains no uniformly accepted method to predict which HER2+ patients are more or less likely to achieve a pathCR. Currently the only standard diagnostic tests to support the addition of trastuzumab are IHC and FISH and neither predicts pathological complete response. As treatment options expand for treatment of HER2 driven tumors, increased assay predictability and specificity will be sought. Here, we seek to identify quantitative measurement of HER2 protein level that are able to specifically select the patients who will most benefit from a trastuzumab containing treatment regime, and those who are not likely to achieve the desired pathCR status.

BrUOG BR-211B (NCT00617942) was a pre-surgical trial for stage II-III HER2+ breast cancer patients led by the Brown University Oncology Group (BrUOG) and the Yale Cancer Center. Patients were treated with q3week carboplatin, weekly nab-paclitaxel and trastuzumab for 18 weeks. In this trial, research biopsies were collected before initiation of treatment and again after a brief "run-in" exposure to either trastuzumab or nab-paclitaxel before patients received their first doses of the entire treatment regimen, allowing the testing of two hypotheses: 1) that the level of HER2 or pHER2 is associated with the likelihood of achieving a pathological complete response; and 2) that short exposure "run-in" treatment with trastuzumab alters the expression of HER2 or pHER2. In this paper, we quantitatively measured the level of HER2 and pHER2 (pY1248) to address these two issues.

## Methods

### BrUOG BR-211B study design

The trial, BrUOG BR-211B (NCT00617942, initiated February 6, 2008), was designed to determine the clinical and pathologic response rates of treatment with q3week carboplatin, weekly nab-paclitaxel and weekly trastuzumab in resectable and unresectable locally advanced breast cancer. Eligibility for the trial included histologically documented adenocarcinoma, female age greater than 18, stage IIA-IIIC disease, no evidence of metastatic disease, no prior systemic therapy, not pregnant or lactating, no baseline neuropathy greater than or equal to grade 2, and HER2 positive defined by IHC 3+ or FISH ratio greater than or equal to 2.0. Baseline biopsies were obtained before the treatment. Randomized "run-in" treatment was given to patients with either a single dose of trastuzumab 6 mg/kg or two weekly doses of nab-paclitaxel 100 mg/m^2^ (determined by the institution at which the patient enrolled on the study). After 9 – 13 days biopsies were repeated. Patients then received trastuzumab 2 mg/kg weekly (patients who received "run-in" nab-paclitaxel were treated with 4 mg/kg for the first week), weekly nab-paclitaxel 100 mg/m2 and carboplatin AUC 6 every 3 weeks for 18 weeks. Pathologic complete response (defined as the absence of residual invasive breast cancer in the breast and axillary nodes) was then assessed at definitive surgery within 6 weeks from the last dose of pre-surgical therapy. Pairs of biopsies (before and after "run-in" treatment) from twenty-seven patients were obtained for biomarker studies (Tables 1 and 2). This trial received ethical approval from both the Yale Human Investigation Committee and the institutional review boards at the participating BrUOG hospitals (Women and Infants Hospital, Rhode Island Hospital, Miriam Hospital, Roger Williams Medical Center and Memorial Hospital of Rhode Island). These review boards approved the consent forms and consent was obtained from each patient prior to enrolling in the trial.

**Table 1:** Cohort description

**Variables**	**n**	**%**
**ER**		
Negative	14	51.9%
Positive	9	33.3%
Unknown	4	14.8%
**PR**		
Negative	18	66.7%
Positive	5	18.5%
Unknown	4	14.8%
**Run-in Treatment**		
Trastuzumab	17	63.0%
Nab-paclitaxel	10	37.0%
**Response**		
PathCR	13	48.1%
No PathCR	10	37.0%
Unknown	4	14.8%

**Table 2:** Association with PathCR

**Variable**	**PathCR**	**No PathCR**	**Chi-Sq**	**p**
**ER**			0.833	0.3613
Negative	7 (35.0%)	5 (25.0%)		
Positive	3 (15.0%)	5 (25.0%)		
**PR**			5.000	0.0253
Negative	10 (50.0%)	6 (30.0%)		
Positive	0 (0.0%)	4 (20.0%)		
**Run-in Treatment**			1.308	0.2528
Nab-paclitaxel	7 (30.4%)	3 (13.1%)		
Trastuzumab	6 (26.1%)	7 (30.4%)		

### Immunofluorescence and AQUA analysis of tissue biopsies

**Figure 1:**
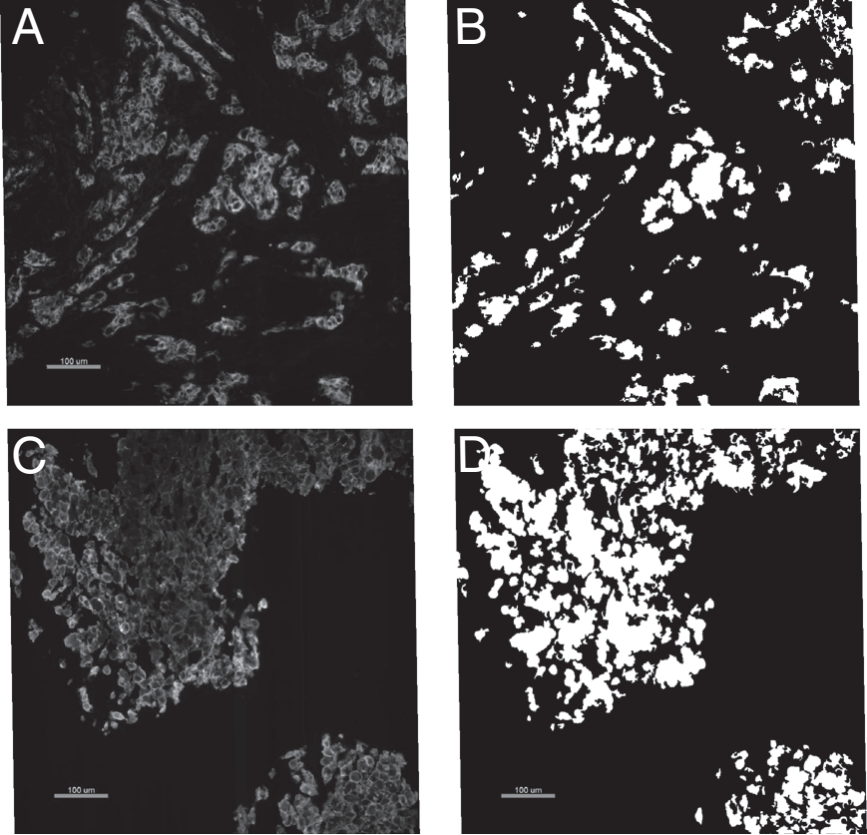
**HER2 and pHER2 staining. A**: A representative image of HER2 expression visualized with CB11 antibody; **B**: Tumor area defined by cytokeratin expression for image **A**; **C**: phopho HER2 (pY1248) expression visualized with the PN2A antibody; **D**: Tumor area defined by cytokeratin for image **C**.

Formalin fixed paraffin embedded tissue biopsies from twenty-seven patients were collected and immediately fixed in formalin to prevent artifact associated with delayed time to fixation. Specimens were then processed, sectioned and deparaffinized in xylene, followed by antigen retrieval at 97°C in pH 6 sodium citric buffer for 20 minutes. Slides were incubated with 0.3% bovine serum albumin in 0.1 M tris-buffered saline with 0.05% Tween-20 to block non-specific binding, then incubated with primary antibodies at 4C overnight. The primary antibodies to HER2 (CB11, Biocare, Concord, MA) and to phospho-HER2 pY1248 (PN2A, LabVision, Fremont, CA) have been previously extensively validated and were only tested for reproducibility before use in our studies. Antibodies diluted 1:1000 and 1:100 respectively showed uniform membrane staining as has been previously described for each (Figure 1). Then the slides were treated with Mouse Envision™ (Dako, Carpinteria, CA) at room temperature for one hour. Cy5 tyramide (NEN Life Science Products, Boston, MA) was then incubated for 10 min at room temperature. Cytokeratin was stained by polyclonal rabbit anti-pan cytokeration (Dako, Carpinteria, CA) and DAPI was then added to define tumor region and subcellular localization required for AQUA analysis.

The algorithm of AQUA analysis is described in detail elsewhere [20,21]. Briefly, regions that contain positive cytokeratin signal were defined as region of interest (ROI), signal of total intensity of a target in each ROI was measured under tumor mask defined by cytokeratin staining and normalized by light source, filter and exposure time, then the total intensity is divided by tumor mask area to generate an AQUA score of each ROI, which is a quantitative measure of the target protein normalized by tumor area. AQUA scores from all of the ROI from each biopsy sample were then averaged to represent the AQUA score for the biopsy.

### Statistical analysis

The statistical calculations were performed using StatView (SAS, Cary, NC). Pathological complete response was used to stratify patients, Fischer’s PLSD was used to compare the mean difference of target biomarkers within each groups. Ordinary least square (OLS) method and paired t-test were used to test if run-in treatment alternated the expression of biomarkers.

## Results

### Association of HER2 level before and after one dose treatment with trastuzumab response

Among the 27 patients, assessment of pathologic response was available for 23. Of these patients, 20 pretreatment biopsies were evaluable, as were 12 of the repeat biopsies after ‘run-in’ treatment. Among the 20 patients with evaluable tissue and known response, 50% [10] had pathCR. The mean baseline HER2 level measured by CB11 antibody in the pathCR group is 10214 AQUA score units, compared with 4766.3 in the no pathCR group (p = 0.0021) (Table 3). This result suggests that a higher HER2 level is associated with pathological complete response. In other studies, we have shown that the lowest AQUA score in a patient with a cancer that was HER2 3+ by immunohistochemical staining was 2111.6 (data not shown) [22] and therefore the mean of HER2 expression in the group with no pathCR is well above the minimum HER2 3+ level. While there was not a substantial change in HER2 levels between the baseline and post-‘run-in’ samples in either group, due to the small number of post-‘run-in’ biopsy samples in the pathCR group the difference in HER2 levels between the pathCR and no pathCR groups for that timepoint no longer reached statistical significance (Table 3).

**Table 3:** Association between the level of HER2 and PathCR

**A**							
	**no-PathCR**			**PathCR**			
	**N**	**Mean**	**SEM**	**N**	**Mean**	**SEM**	**P value**
HER2 AQUA (base)	10	4766.3	1381.1	10	10214.6	639.1	**0.0021**
HER2 AQUA (post)	9	4853.5	1428.0	3	8309.3	1256.0	0.2191
Post/Base AQUA ratio	9	1.09	0.203	2	0.765	0.089	0.4897
**B**							
	**no-Path CR**			**Path CR**			
	**N**	**Mean**	**SEM**	**N**	**Mean**	**SEM**	**P value**
pHER2 AQUA (base)	10	2183.7	472.2	11	2604.9	538.7	0.5669
pHER2 AQUA (post)	9	2482.7	507.2	7	3042.4	1005.5	0.6030
**Post/Base AQUA ratio**	**9**	**1.49**	**0.326**	**6**	**0.919**	**0.145**	**0.1953**

**Figure 2:**
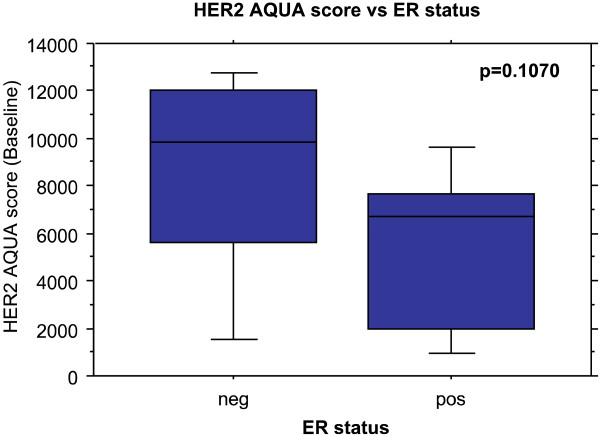
HER2 AQUA score is not statistically significantly correlated with ER status.

Since there is a known inverse association between estrogen receptor (ER) and response in the pre-surgical setting, we evaluated the mean levels of HER2 protein as a function of clinical ER status. The mean AQUA score in ER negative patients was 8615 compared to the 5498 in ER positive patients. This difference is not statistically significant in a two sample mean test (p = 0.1070, Figure 2) in our limited patient population.

**Figure 3:**
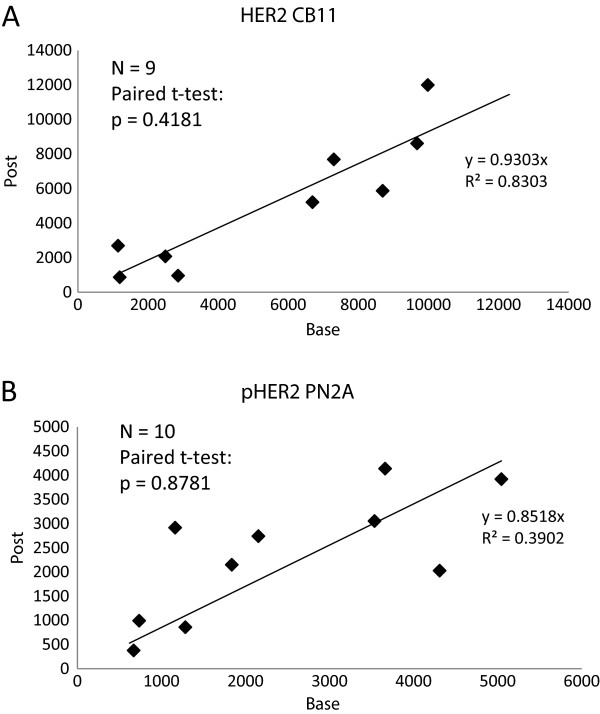
**Biomarker change with one dose of trastuzumab. A**: HER2 expression level change before (base) and after (post) one dose of trastuzumab shown in AQUA scores; **B**: phospho HER2 expression level change before (base) and after (post) one dose of trastuzumab shown in AQUA scores.

Among the 27 patients, 17 were treated with trastuzumab as the "run-in" treatment and 10 were treated with nab-paclitaxel. Nine of 17 trastuzumab treated patients had paired evaluable baseline and post-treatment biopsies. We compared the HER2 level in these 9 pairs. Linear regression with fixed intercept term showed an R^2^ value of 0.83 and a slope term of 0.93 (post-treatment AQUA score unit as the dependent variable). This slope is not statistically significantly different from 1, suggesting that HER2 level is not affected by a single dose of trastuzumab treatment. The result is confirmed by the paired t-test of these 9 pairs of baseline and post-treatment biopsies – the mean of post baseline difference is 449.4 AQUA score units (p = 0.4181) (Figure 3A).

### Association of phospho-HER2 (pY1248) level before and after one dose treatment with response to trastuzumab

For the phospho-HER2 studies, 21 out of 23 biopsies before single dose treatment were evaluable. Also 17 out of 23 biopsies after one dose run-in treatment were evaluable. Among the 21 patients with evaluable tissue and annotated response, 9 had a PathCR (47.3%). The mean of pHER2 level measured by the PN2A antibody in the pathological complete response group was 2768.0 AQUA score units, compared with 2183.7 in the no PathCR group (p = 0.4689) (Table 3). This result suggested that pHER2 level was not associated with pathological complete response. We also tested the mean of pHER2 level after one dose therapy or the pHER2 ratio between post-treatment and baseline biopsies, neither of which was associated with pathological complete response (Table 3).

In the pHER2 evaluable subset, 10 out of 17 trastuzumab run-in treated patients had paired evaluable baseline and post-treatment biopsies. When we compared the pHER2 level in these 10 pairs, linear regression with a fixed intercept term showed an R^2^ value of 0.39 and a slope term of 0.85 (post-treatment AQUA score unit as dependent variable). The slope is not statistically significantly different from 1 (p = 0.212), suggesting that pHER2 level is not altered by the single dose of trastuzumab treatment. This result is confirmed by the paired t-test of the 9 pairs of baseline and post-treatment biopsies – the mean of post baseline difference is 61.3 AQUA score units (p = 0.8781) (Figure 3B).

### No association between HER2 and pHER2 (pY1248)

**Figure 4:**
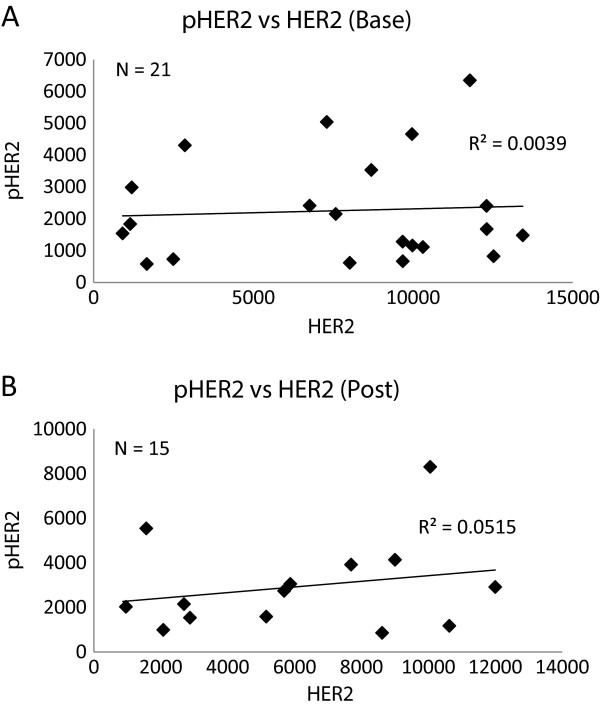
**Association between HER2 and pHER2 level. A**: Association between HER2 and pHER2 level in baseline biopsies, by AQUA score; **B**: Association between HER2 and pHER2 level in post "run-in" treatment biopsies by AQUA score.

We also examined the relationship between HER2 and pHER2 (pY1248) levels. Among baseline biopsies, 21 samples had both evaluable tissues for HER2 and pHER2, and among post-treatment biopsies, 15 samples had evaluable tissues for both HER2 and pHER2. We did not observe an association between HER2 and pHER2 in either baseline biopsies or in post-treatment biopsies (Figure 4).

## Discussion

Few biomarker studies have been able to predict the clinical benefit of the addition of trastuzumab to chemotherapy in the pre-surgical setting, partially because the concept of adding trastuzumab is relatively recent. In addition, patients undergoing pre-surgical therapy typically have limited tissue available for biomarker studies. As a result, studies on pre-surgical therapy tissue are challenging. Furthermore, tissues from the initial core biopsies are small, and often partially or completely exhausted by the diagnostic process. Therefore the availability of tissue for biomarker research in the pre-surgical setting represents a challenge. In order to obtain more tissue, this study was designed to obtain tissue specifically for research by core biopsy at both the initial time point and 10 days after "run in" therapy. This design allowed assessment of biological changes after one dose pre-surgical treatment. While the study is not large, the dedicated tissue collection allowed us to demonstrate that the level of HER2 is higher in patients that respond, and that the levels do not change between the initial biopsy and the post-treatment biopsy.

Biomarker studies have found several biomarkers, such as HER2 level [23] and PI3Kinase Akt pathway activity [24,25], that are associated with response to trastuzumab in adjuvant and metastatic settings. Consistent with that data, in this study, we were able to demonstrate that the higher level of quantitatively measured HER2 is associated with pathCR. However, we did not find an association between pHER2 (pY1248) level and outcome. Increased phospho-HER2 (pY1248) levels have been associated with worse prognosis [26,27], but this data has been hard to confirm due to challenges associated with phospho-epitope stability. In human tumors, the association between the level of phospho-HER2 and outcome remains controversial [28,29]. While our cohort is small, we find no evidence for an association between phospho-HER2 and response to trastuzumab in the pre-surgical setting.

Measurement of phospho-HER2 is challenging since phospho-epitopes are known to be substantially more susceptible to degradation as a function of time to fixation [30,31]. Our recent work (Neumiester et al., in press) and that of others [32] has shown that rapid fixation, as can be achieved by core needle biopsy, is the most effective way to preserve phospho-epitopes. In this study, dedicated core needle biopsy tissues were fixed immediately to minimize artifacts associated with pre-analytic cold ischemic time. Although some previous studies predicted that phospho-HER2 should be associated with pathway activity and potentially with drug response, we believe the phospho-epitopes measured here were accurately determined and they do not support a relationship with response in the pre-surgical setting.

## Conclusions

Overall, this work should be considered as a pilot or proof of concept study. Although limited by small sample size, our data suggests that careful measurement of HER2 levels could provide increased specificity in selection of patients for trastuzumab therapy. As the number of therapies that target the HER2 signaling pathway increases it may be important to select patients less likely to respond to trastuzumab so they can be studied with alternative or combination therapies. Future studies that quantify the levels of HER2 in the pre-surgical setting are needed to confirm this observation.

## Abbreviations

ADCC: Antibody-dependent cell-mediated cytotoxicity; FISH: Fluorescence in situ hybridization; IHC: Immunohistochemistry; OLS: Ordinary least square; OS: Overall survival; PathCR: Pathological complete response; ROI: Region of interest.

## Competing interests

D. Rimm declares a potential conflict of interest since he serves as a consultant to Genoptix, Novaritis and Perkin Elmer. W. Sikov has served on advisory boards for Celgene (on which the study sponsor, Abraxis BioScience, Inc., is now a wholly owned subsidiary). M. Abu-Khalaf, L. Harris, N. Sinclair, H. Cheng, Y. Bai, and V. Bossuyt have no competing interests.

## Authors’ contributions

HC did the quantitative measurements in the lab and the data analysis and wrote the first draft of the manuscript. YB assisted in the quantification of the specimens. WS designed the trial, dealt with clinical issues and problems during the conduct of the trial, oversaw clinical data collection and analysis, and assisted in manuscript preparation. NS assisted with the trial execution at the Brown site, overall clinical data collection and analysis, and assisted in manuscript preparation. VB read the pathology at Yale and did all aspects of the work related to determination of pathologic complete response. MK assisted with the trial execution at the Yale site. LH ran the trial at the Yale site, assisted with data analysis and assisted in manuscript preparation. DR runs the lab in which the work was done, reviewed all of the data collection and analysis and assisted in manuscript preparation. All authors read and approved the final version of the manuscript.

## Pre-publication history

The pre-publication history for this paper can be accessed here:

http://www.biomedcentral.com/1471-2407/14/326/prepub
